# Anxiety, depression, and social connectedness among the general population of eight countries during the COVID-19 pandemic

**DOI:** 10.1186/s13690-022-00990-4

**Published:** 2022-11-18

**Authors:** Di Long, Gouke J. Bonsel, Erica I. Lubetkin, Mathieu F. Janssen, Juanita A. Haagsma

**Affiliations:** 1 Department of Public Health, Erasmus MC, P.O. Box 2040, Rotterdam, 3000 CA The Netherlands; 2 EuroQol Research Foundation, Rotterdam, the Netherlands; 3 Department of Community Health and Social Medicine, CUNY School of Medicine, New York, NY USA; 4 Department of Psychiatry, Section Medical Psychology and Psychotherapy, Erasmus MC, Rotterdam, The Netherlands

**Keywords:** Anxiety, Depression, COVID-19, Social connectedness, Factor analysis

## Abstract

**Background:**

The COVID-19 pandemic affected the mental health of the general population through multiple pathways. The aim of this study was to examine anxiety, depression, self-confidence, and social connectedness among the general population of eight countries during the COVID-19 pandemic, their underlying factors, and vulnerable groups.

**Methods:**

A web-based survey was administered to persons from the general population of China, Greece, Italy, Netherlands, Russia, Sweden, the United Kingdom, and the United States. The survey included the Generalized Anxiety Disorder-7 (GAD-7), Patient Health Questionnaire-9 (PHQ-9) and items on self-confidence, social connectedness, and socio-demographics. Data were analyzed with descriptive statistics, exploratory factor analysis and regression analysis.

**Results:**

Twenty-three thousand six hundred twenty-two respondents completed the survey. Overall, 42% of the total sample had mild to severe anxiety symptoms and 43% had mild to severe depression symptoms. 14% to 38% reported suboptimal ratings in self-confidence, social participation, contact with family and friends, and feeling connected to others. In the exploratory factor analyses, in most countries, one dominant factor had a high influence on GAD-7, PHQ-9 sum scores and self-confidence with eigenvalue (% variance) above 3.2 (53.9%). One less dominant factor had a high influence on social connectedness scores with eigenvalue (% variance) ranging above 0.8 (12.8%). Being younger, female, having chronic conditions, perceived as risky to COVID-19 infection, and feeling not very well protected against COVID-19 were significantly associated with the two underlying factors.

**Conclusions:**

Anxiety, depression, and problems with self-confidence and social connectedness were highly prevalent in the general population of eight countries during the early phase of the COVID-19 pandemic. This highlights the importance of the allocation of additional resources to implement policies to mitigate the impact of the pandemic on mental health.

**Supplementary Information:**

The online version contains supplementary material available at 10.1186/s13690-022-00990-4.

## Introduction

An array of psychosocial effects emerges from the COVID-19 pandemic, through multiple direct and indirect factors, such as fear of contracting or transmitting COVID-19 [1], discontinuation of usual health care and economic factors, such as job loss [2], suspension of education [3, 4], and income uncertainty [5, 6]. Several studies showed an increase in psychological distress and anxiety and depression symptoms in the general population during the COVID-19 pandemic [7–9]. This impact is highest among young people, females, unemployed persons and persons with a chronic illness [7].

Studies determined self-confidence and social connectedness as strong indicators in engaging in protective behaviors[10, 11]. Social connectedness refers to the experience of belonging to a social relationship or network [12]. In the context of COVID-19, protective behaviors (such as mask-wearing, quarantine, social distancing and other lockdown restrictions) prevent or reduce the risk of infection and the fear thereof. However, the imminent impact of the COVID-19 pandemic on the mental health of the general population may be aggravated by the effect of the protective behaviors as they have a detrimental effect on day-to-day communication, physical contact, and social connectedness [13, 14]. Phenomena of psychological distress may be linked bi-directionally to low self-confidence and social connectedness. Lack of social connections and reduced self-confidence are associated with psychological distress [15, 16]. However, anxiety and depression symptoms, such as reduced enjoyment may impair social relations and manifest through low self-confidence [17].

At the early stage of the pandemic, countries have adopted various levels of stringency of government responses against the spread of COVID-19, in response to the number of COVID-19 cases and deaths in each country [18]. Apart from the dynamics of the COVID-19 pandemic and the pre-existing level of anxiety and depression in each population, cross-cultural differences in the experience and conceptualization of anxiety and depression symptoms may explain cross-country differences in the prevalence of anxiety and depression symptoms during the COVID-19 pandemic. Such differences could be reflected in a differential response on targeted questionnaires [19, 20]. This may extend to cross-cultural insensitivity of instruments that are used to assess anxiety and depression symptoms [21, 22].

We conducted a large multi-country study with a set of psychological measures and social connectedness items during the first wave of the pandemic among a set of countries that differed greatly in terms of epidemiological profile of COVID-19 and stringency of the protective restrictions to limit the spread of COVID-19 in the population. The aims of the study were:

1. Assess the prevalence of anxiety and depression symptoms, self-confidence, and social connectedness among the general population in each country and compare them in subgroups relevant to the pandemic such as age and sex;
2. Examine the relationship between anxiety and depression symptoms, self-confidence, and social connectedness, and analyze the factor structure of the instruments used across different countries;
3. Interpret country profiles of psychological and social outcomes, and the relation between them and other risk factors.

## Data and methods

### Study design and population

This study is part of the POPulation health impact of the CORoNavirus disease 2019(COVID-19) pandemic (POPCORN) study. In this cross-sectional study, a web-based survey was administered to a cohort of persons from the general population of eight countries: China, Greece, Italy, the Netherlands, Russia, Sweden, the United Kingdom (UK) and the United States (US).

### Data collection procedure and consent

The participants were recruited by an international market research agency (Dynata) that distributed and launched the questionnaire. Existing internet panels from the eight countries were used, and these samples were designed to be representative of the population aged 18 to 75 years in each country with respect to age and sex (Appendix Fig. 1). The participants were members of the market research agency’s existing voluntary panels. As panel members, the participants had already provided informed consent to participate in online surveys upon registration. Once participating, the data capture system did not allow missing values. Participants received an incentive in the form of cash or points from the market research company upon completion of the survey. Data were anonymized.

### Questionnaire

The questionnaire included questions on demographic and social risk factors, health-related and COVID-19-related risk factors, anxiety symptoms and depression symptoms, self-confidence, social participation, contacts with family and friends, and feeling connected to others. Data were collected from April 22 to May 5, 2020, in China, Greece, Italy, the Netherlands, the UK, and the US, and from May 26 to June 1 in Russia and Sweden.

### Primary outcome measures

The primary outcome measures were anxiety and depression symptoms, self-confidence, contacts with family and friends, social participation and feeling connected to others.

Anxiety symptoms were measured by the Generalized Anxiety Disorder-7 (GAD-7) [23]. The GAD-7 includes seven items that ask the prevalence of anxiety-related symptoms in the past two weeks, such as “How often have you been bothered by not being able to stop or control worrying?”. The ordinal response options range from “Not at all” (“0”) to “Nearly every day” (“3”). The GAD-7 sum score is the sum of the scores of all items and ranges from 0 to 21. By using the cut-off scores of 5, 10, and 15, the GAD-7 can be categorized into four groups: symptoms of minimal anxiety, mild anxiety, moderate anxiety, and severe anxiety.

Depression symptoms were measured by the Patient Health Questionnaire-9 (PHQ-9) [24]. The PHQ-9 includes nine items that measure the prevalence of depression-related symptoms in the past two weeks, such as “How often have you been bothered by little interest or pleasure in doing things?”. The ordinal response options range from “Not at all” (“0”) to “Nearly every day” (“3”). The PHQ-9 sum score is the sum of the scores of each item and ranges from 0 to 27. By using the cut-off scores of 5, 10, 15, and 20, the PHQ-9 can be categorized into five groups: symptoms of minimal depression, mild depression, moderate depression, moderately severe depression, and severe depression. In the study, both sum scores and categories of the GAD-7 and the PHQ-9 were used.

Self-confidence, social participation, contacts with family and friends, and feeling connected to others were part of the EQ-5D-5L bolt-on questions. The EQ-5D-5L is a generic health-related quality of life instrument that consists of five dimensions: mobility, self-care, usual activities, pain/discomfort, and anxiety/depression [25, 26]. EQ-5D-5L bolt-on questions are items that can be added to the EQ-5D-5L and that have the same format as the EQ-5D-5L items [27]. The self-confidence bolt-on was previously developed [28] whereas the remaining three bolt-on questions were developed for the current study. Self-confidence and social participation were measured by the presence of problems. The ordinal response options to self-confidence are “I have no problems with self-confidence” (“1”), “I have slight problems with self-confidence” (“2”), “I have moderate problems with self-confidence” (“3”), “I have severe problems with self-confidence” (“4”), and “I have extreme problems with self-confidence” (“5”). The ordinal response options to social participation are similar as self-confidence, ranging from “I have no problems with social participation” (“1”) to “I have extreme problems with social participation” (“5”). Contact with family and friends and feeling connected to others were measured by self-rating. The ordinal response options of contact with family and friends are “Very good” (“1”), “Good” (“2”), “Fair” (“3”), “Bad” (“4”), and “Very bad” (“5”), and the ordinal response options of feeling connected are “I feel very well connected to others” (“1”), “I feel well connected to others” (“2”), “I feel moderately connected to others” (“4”), “I feel slightly connected to others” (“4”), and “I feel not connected to others, alone” (“5”).

These outcome variables were grouped into two concepts: internal states (GAD-7, PHQ-9, and self-confidence) and social connectedness (social participation, contact with family and friends, and feeling connected to others).

### Other measures of respondent characteristic

The following risk factors were included: age, sex, the highest level of education achieved, occupational status, income, living situation, self-perception of COVID-19 risk, COVID-19 disease status, number of chronic diseases, perceptions of being protected against COVID-19, chronic disease status and quality of health care (i.e., access to health care).

Categorization of education and income can be found in the appendix. Chronic disease status was measured by the presence of up to 10 chronic conditions (asthma and chronic bronchitis, severe heart disease, stroke, diabetes, severe back complaints, arthrosis, rheumatism, cancer, memory problems, and/or other problems). The number of chronic diseases was categorized into three groups: “zero”, “one”, “two”, and “three or more”.

Quality of care was derived from the experience of the respondent during their last outpatient visit following the World Health Organization (WHO) responsiveness measures [29]. Experience on access to health care was scored with ordinal response options ranged from “very good/always good” to “very bad/never good” (Appendix).

The questionnaire was translated into the main official language of each country using translation software and subsequently translated back into English, except when case validated translated versions of the instruments were available. Bilingual native speakers verified the translations independently.

### Statistical analysis

Descriptive analyses were performed for sociodemographic data, anxiety and depression symptoms, self-confidence, social participation, contacts with family and friends, and feeling connected to others. Percentage distributions were calculated for GAD-7 and PHQ-9 by ten-year age groups in each country. For self-confidence, contacts with family and friends, social participation and feeling connected to others, percentage distributions were calculated by country. We then calculated rate ratios between females and males of the prevalence of anxiety and depression symptoms among each 10-year age group in each country. Self-confidence, social participation, contacts with family and friends, and feeling connected to others were also assessed by country and age category.

After very high correlations were found between several factors and between the GAD-7 and PHQ-9 as well as high Cronbach's alpha (Appendix), exploratory factor analysis was performed in each country among a selection of observed variables: GAD-7 and PHQ-9 sum score, self-confidence, contact with family and friends, social participation, and feeling connected to others to test whether homogeneous constructs was underlying the data. Factor analysis was also performed among GAD-7 and PHQ-9 item scores.

Our data were tested to be suitable for factor analysis by Kaiser–Meyer–Olkin measure of sampling adequacy and Bartlett’s test of Sphericity [30]. Principal axis factoring was chosen as the extraction method. Because we were interested in the latent factor that underlines the data and since multivariate normality was violated in our data, the interrelationship was studied by principal axis factoring [31]. The number of factors extracted was examined based on the Scree test and parallel analysis. An oblique rotation (Promax rotation, kappa set at 4) was used. A set of factor matrices, pattern matrix, structure matrices, and factor correlation matrices were generated. If factors correlations were low, an orthogonal rotation would be chosen before re-running the rotation. Factor loadings above 0.4 were considered as interpretable [32]. The exploratory factor analysis was performed for all countries pooled and for each country in order to determine if there were differences between countries in the latent concepts observed.

Factor scores of each factor for individual respondents in each country were calculated by a regression method. In the next step, the individual factor scores were used as outcome variables, and linear multivariable regression analyses were performed on the risk factors. The likelihood ratio test was used, and for overall *p* values, the significance level was set at 0.05.

All statistical analyses were carried out using R version 4.0.5 and SPSS version 25.

## Results

### Study population

In total, 26,503 persons indicated that they wanted to participate, of whom 23,622 (89.1%) completed the survey. 23,513 (99.5%) were included in the study (Appendix).

Table 1 shows the characteristics of the respondents by country. The median age of the respondents ranged from 34 (IQR: 15) in Chinese sample to 49 (IQR: 29) in the Dutch sample. Slightly more than half of the respondents were female (50% in Russian sample to 56% in Chinese sample). 44% (Swedish sample) to 84% (Chinese sample) of the respondents reported no chronic conditions and most respondents reported not being infected with COVID-19 (> 78%). For each country, the COVID-19 incidence and mortality rates and government response against COVID-19, as measured by the stringency index by country, are summarized in Appendix Table 2.

**Table 1:** Sociodemographic and health-related characteristics of respondents from eight countries during the early stage of COVID-19 pandemic

	**China** ***N* = 3226**	**Greece** ***N *= 959**	**Italy** ***N* = 3210**	**Netherlands** ***N* = 3293**	**Russia** ***N* = 3166**	**Sweden** ***N* = 3209**	**UK** ***N* = 3230**	**US** ***N* = 3220**	**Total** ***N* = 23,513**
***Age***									
Median (IQR)	34.0(15.0)	39.0(20.0)	43.0(22.0)	49.0(29.0)	40.0(22.8)	48.0(28.0)	44.0(27.0)	46.0(27.0)	42.0(25.0)
Mean (SD)	35.9(11.7)	40.3(13.2)	44.0(14.2)	47.9(16.6)	40.7(14.0)	47.6(16.3)	45.5(15.9)	46.5(16.1)	43.9(15.5)
***Age group***									
18–24 yrs	16%	16%	8.6%	10%	17%	9%	10%	11%	12%
25–34 yrs	35%	21%	20%	16%	22%	17%	20%	17%	21%
35–44 yrs	26%	24%	26%	16%	19%	17%	22%	19%	21%
45–54 yrs	14%	21%	21%	18%	21%	19%	16%	18%	18%
55–64 yrs	8.0%	14%	14%	19%	15%	17%	16%	17%	15%
65–75 yrs	1.1%	3.4%	11%	21%	5.1%	20.2%	17%	18%	13%
***Sex***									
Male	44%	48%	48%	48%	50%	47%	48%	44%	47%
Female	56%	52%	52%	52%	50%	53%	52%	56%	53%
***Education level***									
High	59%	61%	42%	44%	52%	59%	61%	58%	54%
Middle	31%	35%	45%	30%	46%	31%	37%	35%	36%
Low	10%	3.9%	14%	25%	1.9%	11%	2.3%	6.9%	10%
***Occupation status***									
Employed	84%	53%	58%	51%	63%	49%	60%	52%	59%
Student	6.6%	9.5%	6.9%	7.4%	8.0%	7.2%	3.1%	3.0%	6.2%
Unemployed	4.7%	28%	23%	12%	15%	14%	12%	16%	14%
Retired	3.8%	8.0%	12%	20%	12%	24%	18%	21%	15%
Unable to work	0.4%	1.7%	0.9%	10%	1.5%	5.5%	7.1%	8.1%	4.7%
***Household income***									
High	23%	31%	13%	24%	15%	21%	20%	28%	21%
Middle	52%	29%	55%	42%	64%	48%	48%	49%	50%
Low	22%	29%	22%	17%	14%	19%	24%	17%	20%
Unwilling to tell	2.7%	11%	11%	16.9%	7.0%	12%	8.4%	5.5%	9.1%
***Chronic conditions***									
0	80%	59%	62%	50%	56%	44%	57%	50%	57%
1	13%	31%	27%	31%	30%	33%	27%	31%	28%
2	3.9%	6.5%	7.4%	11.2%	9.5%	14.1%	10%	11%	9.4%
3 or more	2.9%	2.8%	4.2%	7.9%	5.0%	9.0%	6.0%	9.1%	6.2%
***Self-perception of risk to COVID-19***									
No	78%	61%	63%	57%	57%	57%	61%	57%	61%
Yes	22%	39%	37%	43%	43%	43%	39%	43%	39%
***COVID-19 status***									
Not infected	96%	94%	90%	86%	94%	78%	85%	84%	88%
Infected but recovered	0.2%	0.1%	0.5%	0.9%	0.5%	1.2%	0.9%	1.4%	0.8%
Maybe infected	3.5%	6.3%	10%	13%	5.7%	20%	14%	13%	11%
Infected	0.1%	0.1%	0.0%	0.3%	0.1%	0.3%	0.3%	1.4%	0.4%
***Living situation***									
Living alone	7.7%	14%	10%	26%	13%	31%	20%	22%	18%
Living alone with children	3.2%	7.3%	5.9%	6.6%	6.3%	9.0%	8.6%	7.9%	6.8%
Living with other adults	24%	39%	41%	40%	36%	35%	41%	40%	37%
Living with other adults and children	63%	38%	41%	25%	41%	22%	28%	26%	35%
Other	2.0%	2.0%	1.8%	1.6%	4.8%	2.1%	2.3%	4.3%	2.7%
***Feeling protected against COVID-19***									
Very well	60%	41%	15%	14%	14%	16%	23%	29%	25%
Well	34%	41%	44%	52%	38%	35%	38%	34%	39%
Reasonably	5.6%	15%	35%	30%	36%	35%	34%	31%	29%
Insufficiently	0.4%	2.8%	6.4%	4.7%	12.8%	13.9%	5.1%	5.7%	6.8%
***Social participation***									
No problems	82%	73%	67%	69%	70%	51%	61%	59%	66%
Slight problems	15%	19%	21%	18%	21%	25%	21%	20%	20%
Moderate problems	2.0%	5.5%	7.9%	7.8%	6.9%	13%	10%	12%	8.4%
Severe problems	0.6%	1.5%	2.4%	3.4%	1.9%	7.3%	4.1%	4.3%	3.3%
Extreme problems	0.4%	0.9%	1.8%	1.6%	0.8%	3.7%	3.6%	4.5%	2.3%
***Contact with family and friends***									
Very good	64%	49%	31%	33%	35%	33%	29%	37%	38%
Good	27%	34%	44%	44%	46%	34%	39%	37%	39%
Fair	8.9%	14%	17%	18%	16%	21%	24%	20%	18%
Bad	0.5%	1.5%	4.6%	3.5%	1.9%	8.3%	5.6%	3.8%	3.9%
Very bad	0.2%	0.8%	2.7%	1.3%	0.4%	3.1%	2.6%	2.3%	1.8%
***Feeling connected with others***									
Very well	52%	49%	28%	34%	16%	25%	30%	35%	32%
Well	24%	33%	38%	40%	25%	25%	29%	27%	30%
Moderately	14%	12%	20%	16%	40%	25%	21%	19%	22%
Slightly	6.4%	4.0%	7.5%	5.9%	12%	19%	13%	12%	11%
Not at all	3.1%	1.8%	5.6%	4.1%	7.6%	6.3%	7.0%	6.7%	5.6%
***Self-confidence***									
No problems	67%	50%	53%	59%	41%	48%	47%	52%	53%
Slight problems	25%	33%	27%	25%	36%	25%	26%	24%	27%
Moderate problems	5.2%	9.2%	13%	9.4%	16%	15%	15%	13%	12%
Severe problems	1.7%	5.7%	4.5%	4.3%	5.3%	8.3%	6.6%	6.2%	5.3%
Extreme problems	0.5%	1.3%	1.8%	1.7%	2.5%	3.6%	4.4%	4.8%	2.7%
***Last outpatient visit***									
> 3 months ago	72%	67%	64%	66%	75%	61%	69%	59%	66%
1 to 3 months ago	15%	23%	26%	21%	15%	17%	21%	24%	20%
1 to 4 weeks ago	9.0%	6.8%	6.0%	7.3%	5.7%	13%	6.7%	10%	8.3%
Last week	4.2%	3.4%	3.5%	5.7%	4.8%	9.5%	3.5%	7.0%	5.4%
***Experience with access to healthcare***									
Very good/Always good	45%	31%	30%	38%	19%	31%	34%	51%	35%
Good/Usually good	41%	38%	45%	46%	41%	37%	39%	33%	40%
Fair/Sometimes good	12%	20%	19%	13%	25%	23%	21%	13%	18%
Bad/Usually not good	1.4%	8.2%	4.2%	2.9%	11%	7.0%	5.0%	2.6%	4.9%
Very bad/Never good	0.2%	2.6%	1.2%	0.6%	4.6%	2.3%	1.5%	1.0%	1.6%

*Note*: For Greece and Russia, income represents individual monthly income, for the rest it represents annual household income

### Anxiety and depression symptoms

**Fig. 1:**
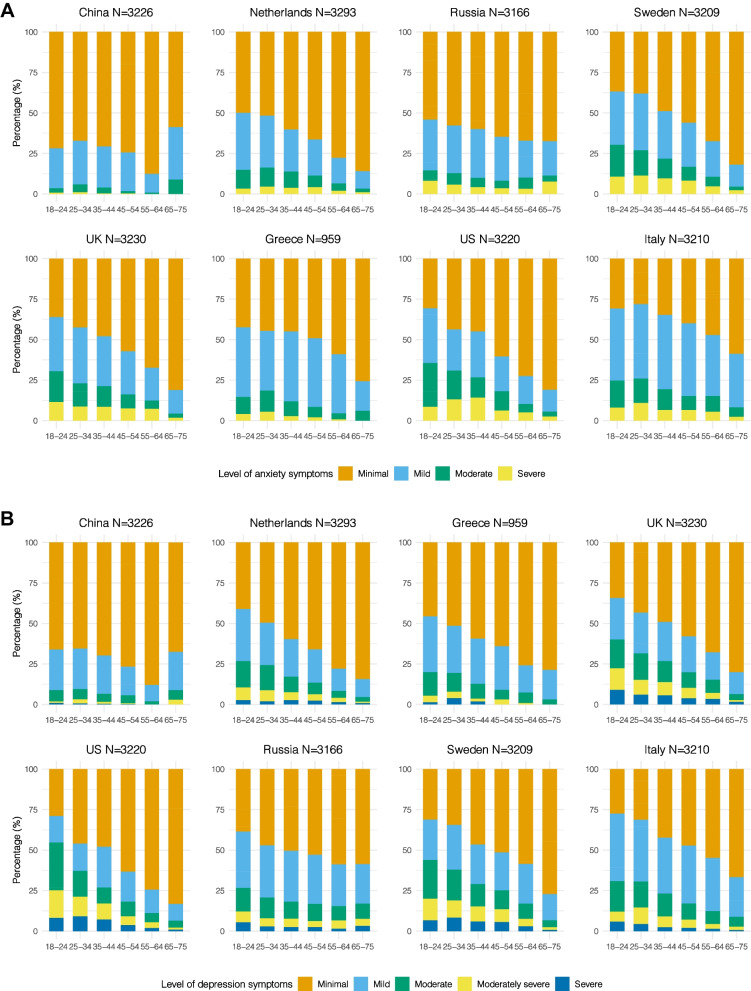
Symptoms of anxiety (**A**) and depression (**B**) by age groups in eight-country samples

Overall, 42% of the total sample had mild to severe anxiety symptoms and 43% had mild to severe depression symptoms. Prevalence of mild to severe anxiety and depression symptoms was lowest for respondents from Chinese sample (anxiety: 29%; depression: 30%) and highest for respondents from Italian sample (anxiety: 61%; depression: 56%) (Fig. 1).

**Fig. 2:**
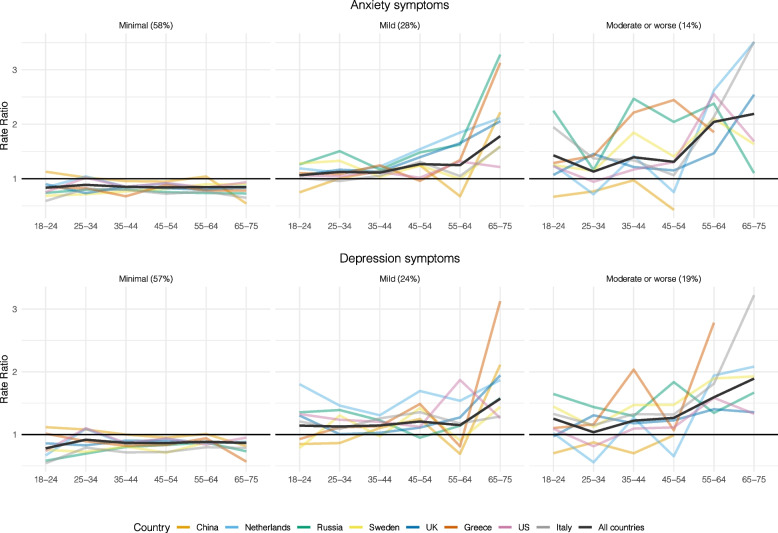
The ratio of anxiety and depression prevalence between sex according to age groups

For all respondents, except for Chinese respondents, the prevalence of mild to severe anxiety and depression symptoms decreased with increasing age. Female respondents reported more anxiety and depression prevalence compared to males (Fig. 2).

### Self-confidence

**Fig. 3:**
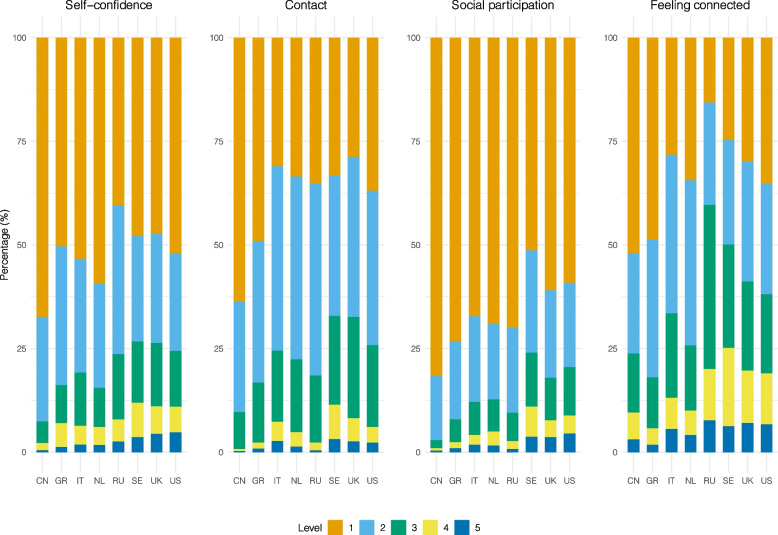
Self-confidence and social connectedness variables in the eight countries. Note to figure: level 1–5 refers to “very good” to “very bad” for contact with family and friends, “no problems” to “extreme problems” for social participation and self-confidence, and “very well” to “not” for feeling connected to others. “CN” to “US” represents “China”, “Greece”, “Italy”, “Russia”, “Sweden”, “the UK” and “the US”

In general, 20% of all respondents reported having moderate to extreme problems with self-confidence (Fig. 3). Respondents in China reported the least problems (7%) and respondents in Sweden reported the most problems (23%) with self-confidence. Across countries, there was a general decrease in reporting problems with increasing age. Subtle differences were found between females and males, except that 21% of males in the UK and Swedish samples reported moderate to extreme problems compared to 31% females in the UK sample and 32% females in Swedish sample reporting problems with self-confidence (data not shown).

### Social connectedness

Of the total sample, 14% reported having moderate to extreme problems with social participation, 23% reported fair to very bad contact with family and friends, and 38% reported feeling moderately to not connected to others (Fig. 3). Almost no one reported having moderate to extreme problems with social participation among Chinese respondents (3%) and more than half of the respondents reported feeling moderately to not connected to others among Russia respondents (60%). There is a general decrease in reporting problems with social participation, feeling connected to others and contacts with family and friends with increasing age and subtle differences between females and males (data not shown).

**Fig. 4:**
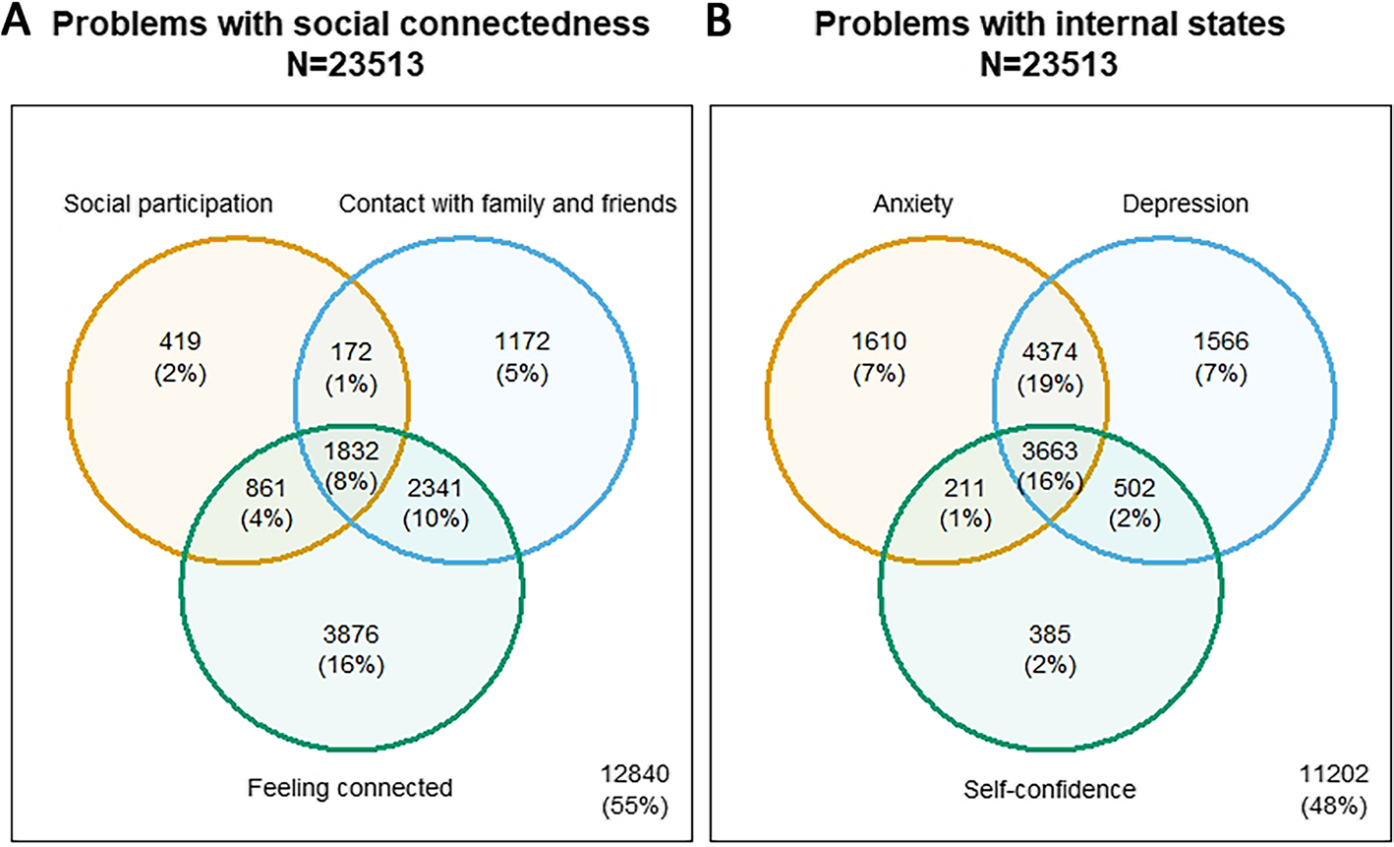
Problems with internal states and social connectedness variables in eight countries. Note to figure: Problems with internal states refer to worse than “mild symptoms of anxiety” and “mild symptoms of depression” (from the levels “minimal” to “extreme”) and worse than “moderate problems with confidence” (from the levels “no problems” to “extreme problem”). Problems with social connectedness refer to worse than “moderate problems with social participation” (from the levels “no problems” to “extreme problem”), “fair contact with family and friends” (from the levels “very good” to “very bad”) and feeling “moderately connected to others” (from the levels “very well” to “not”)

Respondents that reported anxiety and depression symptoms frequently also reported problems with self-confidence. Similar overlap was also found between social participation contacts with family and friends and feeling connected to others (Fig. 4).

### Factor analysis

In Table 2, factor analysis of all countries pooled showed two interpretable factors, which we coined: “internal states” (F1) and “social connectedness” (F2), with eigenvalue (% of variance explained) 3.6 (60.5%) and 0.8 (13.8%). Repeating the factor analysis for each of the 8 countries separately showed very similar results and patterns, except for China and Greece. In Greece, the signs of the coefficients were the same, but the factor structures were reversed. In China, a third factor (F3) was considered. We interpreted F3 in China as (problems with) “objective” social participation, separately from the “feeling” of connectedness.

**Table 2:** Influence of underlying factors on internal states and social connectedness variables, and correlations between factors

*Factor loading*	F1 (Internal states)							F2	F2 (social connectedness)							F1	F3 (partici- pation)	F1	F2
	CN	IT	NL	RU	SE	UK	US	GR	CN	IT	NL	RU	SE	UK	US	GR	CN	All	All
GAD score	**0.89**	**0.82**	**0.92**	**0.87**	**0.95**	**0.93**	**0.90**	**0.86**	0.10	0.01	-0.06	-0.07	-0.07	-0.04	-0.03	-0.08	-0.09	**0.90**	-0.04
PHQ score	**0.93**	**1.00**	**0.93**	**0.92**	**0.90**	**0.97**	**1.00**	**0.86**	-0.08	-0.04	0.02	0.00	0.04	-0.03	-0.06	0.08	0.12	**0.95**	0.00
Self-confidence	0.11	**0.42**	**0.54**	0.38	**0.48**	**0.48**	**0.53**	0.32	0.13	0.35	0.26	**0.42**	0.31	0.35	0.30	**0.45**	**0.55**	**0.43**	0.37
Social participation	-0.03	0.10	0.21	0.19	0.20	0.26	0.29	-0.01	0.02	**0.64**	**0.51**	**0.45**	**0.55**	**0.50**	**0.47**	**0.66**	**0.76**	0.19	**0.55**
Contact with others	0.15	0.03	0.02	0.09	0.13	0.00	-0.01	0.08	**0.48**	**0.62**	**0.66**	**0.59**	**0.58**	**0.70**	**0.70**	**0.74**	0.12	0.04	**0.67**
Feeling connected	-0.04	-0.07	-0.05	-0.14	-0.10	-0.07	-0.06	-0.08	**0.79**	**0.86**	**0.89**	**0.75**	**0.89**	**0.91**	**0.88**	**0.97**	0.01	-0.09	**0.86**
Eigenvalue	3.6	3.5	3.6	3.2	3.7	3.8	3.7	0.9	0.7	0.9	0.9	0.9	0.8	0.9	0.9	3.6	0.6	3.6	0.8
% Variance	60.0	57.7	60.6	53.9	62.1	62.5	61.3	15.2	11.9	15.2	14.2	14.8	12.8	14.2	15.0	60.3	10.1	60.5	13.8
*Correlation*		IT	NL	RU	SE	UK	US	GR	All		CN								
		F2									F2	F3							
F1		0.64	0.67	0.67	0.69	0.66	0.64	0.64	0.68		0.65	0.73	F1						
												0.76	F2						

*Note*: Pattern matrix is presented here as factor loading. F1, F2 and F3 represent Factor 1, Factor 2 and Factor 3

As for the factor analysis on the GAD-7 and PHQ-9 items, three or four highly correlated factors were considered for each country, with one factor having high loadings on all GAD-7 items and the other factors on PHQ-9 items. There were no factors with high loading on both GAD-7 and PHQ-9 items in most countries (Appendix).

### Associations between factors internal states and social connectedness and other (non-)health-related risk factors

Table 3 presents country-specific multivariable linear regression results where factor scores were the outcome variable. Age and living status showed a consistent across-country positive association with both factor scores. Older age strongly contributed to positive internal states and less to social connectedness. Not living alone is related to social connectedness. Across countries, coefficients were similar in size and sign, but statistical significance was fluctuating.

**Table 3:** Factors to non-health and health-related risk factors during the early stage of the COVID-19 pandemic

	**Internal states**								**Social connectedness**								**F3**
	CN	GR	IT	NL	RU	SE	UK	US	CN	GR	IT	NL	RU	SE	UK	US	CN
Intercept	0.36	0.23	0.19	0.05	0.42	0.38	0.40	-0.01	0.42	0.23	0.26	0.35	0.44	0.77	0.73	0.56	0.37
***Age group***	< 0.001	< 0.001	< 0.001	< 0.001	< 0.001	< 0.001	< 0.001	< 0.001	< 0.001	< 0.001	< 0.001	< 0.001	< 0.001	< 0.001	< 0.001	< 0.001	< 0.001
25–34 yrs	0.04	0.11	0.17	0.14	0.13	0.07	0.13	0.18	-0.07	0.09	0.13	0.06	0.09	-0.15	0.02	0.05	0.01
35–44 yrs	0.15	0.30	0.38	0.33	0.29	0.27	0.27	0.30	0.05	0.26	0.19	0.07	0.21	-0.06	-0.01	0.08	0.14
45–54 yrs	0.24	0.47	0.57	0.51	0.43	0.47	0.51	0.59	0.02	0.28	0.33	0.18	0.32	0.06	0.05	0.14	0.20
55–64 yrs	0.48	0.73	0.74	0.82	0.58	0.74	0.67	0.79	0.23	0.56	0.38	0.36	0.40	0.27	0.16	0.23	0.32
65–75 yrs	0.44	0.87	0.78	0.90	0.61	0.89	0.80	0.87	0.25	0.63	0.43	0.41	0.56	0.43	0.33	0.26	0.33
***Sex***	*0.53*	*0.071*	< *0.001*	< *0.001*	< *0.001*	< *0.001*	< *0.001*	*0.15*	*0.51*	*0.062*	*0.50*	*0.41*	*0.23*	*0.30*	*0.21*	*0.46*	*0.28*
Female	-0.02	-0.10	-0.19	-0.10	-0.14	-0.14	-0.10	-0.04	-0.02	0.11	-0.02	-0.02	0.03	-0.03	0.03	-0.02	-0.03
***Education level***	< *0.001*	*0.61*	*0.001*	*0.55*	*0.049*	*0.41*	*0.66*	*0.055*	< *0.001*	*0.75*	*0.001*	*0.058*	*0.082*	*0.21*	*0.50*	*0.75*	< *0.001*
Middle education	0.17	-0.05	0.09	0.01	0.07	0.04	-0.03	-0.05	0.14	0.04	0.06	0.04	0.06	0.04	-0.03	-0.02	0.14
Low education	0.13	-0.10	0.16	-0.03	0.16	0.01	-0.02	-0.12	0.14	0.08	0.18	0.09	0.10	-0.04	0.02	-0.03	0.09
***Occupation status***	< *0.001*	*0.60*	< *0.001*	< *0.001*	< *0.001*	< *0.001*	< *0.001*	< *0.001*	< *0.001*	*0.005*	< *0.001*	< *0.001*	< *0.001*	< *0.001*	< *0.001*	< *0.001*	< *0.001*
Student	-0.17	-0.11	0.00	-0.10	-0.23	-0.09	-0.15	-0.05	-0.35	-0.28	-0.03	-0.28	-0.17	-0.25	-0.15	-0.16	-0.30
Unemployed	-0.27	-0.09	-0.19	-0.26	-0.17	-0.27	-0.25	-0.20	-0.48	-0.16	-0.25	-0.31	-0.22	-0.33	-0.25	-0.30	-0.44
Retired	-0.03	-0.06	0.15	-0.03	0.15	-0.06	0.05	0.04	-0.15	-0.17	0.10	0.01	0.04	-0.18	0.00	-0.01	0.02
Unable to work	-0.76	-0.19	-0.37	-0.32	-0.43	-0.35	-0.41	-0.13	-1.40	-0.61	-0.83	-0.41	-0.54	-0.52	-0.49	-0.33	-1.18
***Household income***	*0.011*	*0.49*	*0.056*	*0.007*	*0.33*	*0.023*	< *0.001*	< *0.001*	*0.006*	*0.62*	*0.43*	*0.17*	*0.088*	*0.026*	< *0.001*	*0.41*	< *0.001*
Middle income	0.06	-0.05	0.04	0.05	-0.05	-0.07	-0.18	0.09	0.03	-0.03	-0.02	-0.01	-0.07	-0.11	-0.21	-0.04	-0.03
Low income	-0.06	0.03	-0.02	0.04	-0.10	-0.14	-0.22	0.17	-0.07	0.07	0.02	-0.03	-0.15	-0.06	-0.33	-0.01	-0.15
Unwilling to tell	-0.04	0.08	0.12	0.16	-0.09	-0.01	-0.07	0.23	-0.17	0.03	0.06	0.07	-0.07	-0.07	-0.13	0.04	-0.17
***Chronic conditions***	< *0.001*	< *0.001*	< *0.001*	< *0.001*	< *0.001*	< *0.001*	< *0.001*	< *0.001*	< *0.001*	< *0.001*	< *0.001*	< *0.001*	< *0.001*	< *0.001*	< *0.001*	< *0.001*	< *0.001*
1 dis	-0.52	-0.47	-0.32	-0.32	-0.34	-0.40	-0.37	-0.33	-0.45	-0.42	-0.32	-0.26	-0.25	-0.39	-0.28	-0.28	-0.47
2 dis	-0.91	-0.66	-0.64	-0.59	-0.65	-0.58	-0.59	-0.59	-0.59	-0.75	-0.45	-0.47	-0.49	-0.51	-0.53	-0.58	-0.80
3 or more	-1.38	-1.48	-1.02	-0.82	-1.09	-0.93	-0.98	-1.00	-0.97	-1.16	-0.83	-0.62	-0.77	-0.83	-0.75	-0.78	-1.23
***Self-perception of risk to COVID-19***	< *0.001*	*0.005*	< *0.001*	< *0.001*	< *0.001*	< *0.001*	< *0.001*	< *0.001*	< *0.001*	*0.30*	*0.084*	*0.19*	< *0.001*	*0.66*	*0.41*	*0.33*	< *0.001*
Yes	-0.33	-0.16	-0.20	-0.14	-0.17	-0.15	-0.12	-0.24	-0.12	-0.06	-0.06	-0.05	-0.12	-0.01	-0.03	-0.03	-0.20
***Living situation***	*0.88*	*0.033*	*0.053*	*0.017*	< *0.001*	*0.15*	< *0.001*	*0.11*	*0.001*	*0.086*	*0.015*	< *0.001*	< *0.001*	< *0.001*	*0.002*	*0.003*	*0.11*
Living alone with children	0.08	0.18	-0.12	-0.05	0.16	-0.06	-0.11	0.03	0.17	0.13	0.12	0.14	0.17	0.12	0.06	0.15	0.05
Living with other adults	0.00	0.21	0.06	0.10	0.18	0.04	0.11	0.09	0.01	0.13	0.13	0.17	0.17	0.13	0.10	0.11	0.02
Living with other adults and children	0.01	0.18	0.01	0.07	0.24	-0.02	0.07	0.08	0.12	0.21	0.16	0.18	0.29	0.16	0.17	0.15	0.09
Other	0.07	-0.17	-0.03	-0.05	0.19	0.15	0.16	0.12	-0.10	-0.13	-0.04	0.06	0.12	0.26	0.14	0.04	-0.05
***Feeling protected against COVID-19***	< *0.001*	< *0.001*	< *0.001*	< *0.001*	< *0.001*	< *0.001*	< *0.001*	< *0.001*	< *0.001*	< *0.001*	< *0.001*	< *0.001*	< *0.001*	< *0.001*	< *0.001*	< *0.001*	< *0.001*
Well	-0.29	-0.17	-0.05	0.00	-0.13	0.00	-0.12	0.06	-0.33	-0.25	-0.13	-0.15	-0.22	-0.16	-0.20	-0.20	-0.26
Reasonably	-0.56	-0.48	-0.20	-0.20	-0.37	-0.23	-0.36	-0.12	-0.69	-0.55	-0.24	-0.40	-0.49	-0.43	-0.48	-0.47	-0.57
Insufficiently	-0.97	-0.72	-0.54	-0.42	-0.68	-0.50	-0.87	-0.59	-0.83	-0.73	-0.56	-0.56	-0.67	-0.62	-0.80	-0.82	-0.78
***Experience with access to healthcare***	< *0.001*	< *0.001*	< *0.001*	< *0.001*	< *0.001*	< *0.001*	< *0.001*	< *0.001*	< *0.001*	< *0.001*	< *0.001*	< *0.001*	< *0.001*	< *0.001*	< *0.001*	< *0.001*	< *0.001*
Good/Usually good	-0.28	-0.12	-0.25	-0.15	-0.20	-0.09	-0.11	-0.17	-0.29	-0.12	-0.27	-0.21	-0.21	-0.18	-0.17	-0.27	-0.24
Fair/Sometimes good	-0.53	-0.28	-0.48	-0.56	-0.23	-0.24	-0.31	-0.39	-0.62	-0.39	-0.48	-0.55	-0.27	-0.35	-0.43	-0.43	-0.54
Bad/Usually not good	-0.53	-0.37	-0.68	-0.56	-0.49	-0.51	-0.48	-0.73	-0.70	-0.35	-0.71	-0.61	-0.46	-0.51	-0.57	-0.76	-0.49
Very bad/Never good	-1.13	-0.60	-0.92	-0.78	-0.66	-0.48	-0.40	-0.95	-1.34	-0.54	-0.99	-0.60	-0.58	-0.61	-0.66	-1.03	-1.68

*Note*: F3 represents objective social participation. Reference group was age 18–25 yrs., male, high-educated, employed, had high income, no chronic conditions, no perceived risk to COVID-19, living alone, was feeling very well protected against COVID-19 and had very good/always good experience on access to healthcare

## Discussion

### Prevalence of anxiety and depression

Our study showed that anxiety and depression symptoms are highly prevalent in most of the sample population of eight countries during the early phase of the COVID-19 pandemic. Several studies found a similarly high prevalence of anxiety and depression symptoms around the same period [6, 33–38], even when different instruments and cut-off scores were used.

In our study, prevalence rates of anxiety and depression were lowest in China and highest in Italy. This may be due to the spread of the COVID-19 infection during the time of data collection. At the time of data collection, the epidemic reached the end of its first peak in China, whereas it was still at its pinnacle in Italy. Stringency of government protective measures against the spread of COVID-19 may contribute to the difference in prevalence of anxiety and depression symptoms. Previous studies showed that a higher level of stringency of government protective measures against the spread of COVID-19 was associated with higher psychological distress [39, 40]. Another explanation for differences in the prevalence of anxiety and depression symptoms may be found in baseline differences. In a WHO report from 2017, China was estimated to have a (slightly) lower percentage of people with anxiety and depressive disorders than Italy, and the south-east Asia region overall had a similar percentage of depressive disorder and a lower percentage of anxiety disorder than the European region [41].

Furthermore, in our study, a universal age pattern in most of the countries stood out, where the younger the age the more prevalent anxiety and depression symptoms. Previous studies have demonstrated inconsistent relations between age and anxiety and depression symptoms/disorders throughout the life course [41–46]. This observed age pattern may be resulting from the development of the nature of anxiety and depression development with age, or it may even be resulting from a cohort effect [47, 48]. It could also be that the public health measures against COVID-19 posed a more profound mental burden on younger people compared to their older counterparts, due to restrictions of such as education and employment and possible fear of missing out on emerging relationships, traditional milestones and rites of passage and opportunities for advancement in life [49–52].

Our study further confirmed a higher prevalence of anxiety and depression among females compared to their male counterparts and this difference in prevalence by gender increased with increasing age [53, 54].

### Factors “Internal states” and “Social connectedness”

In our study, more than 80% of respondents who met the criteria for mild to severe anxiety also met the criteria for mild to severe depression. The co-occurrence of anxiety and depression symptoms was further confirmed by a strong latent concept in factor analysis. While the prevalence of anxiety and depression varied between countries, the latent concept was unanimous across countries. The co-occurrence of anxiety and depression symptoms is frequently observed [55, 56].

In addition, we found that self-confidence was also explained by the factor internal states in the samples from several countries. This is partly understandable as low self-confidence frequently co-occurs with depression [57–59].

Social connectedness was also found to be highly correlated to internal states in our study. Previous studies suggested that anxiety and depression are often characterized by perceived social disconnection [60]. Social connectedness is even hypothesized to mediate the relationship between social support and depression [61]. In the context of the COVID-19 pandemic, social connectedness may be linked to anxiety and depression symptoms through several pathways. For example, restriction measures that reduced social connectedness could trigger anxiety and depression [62, 63] or, conversely, anxiety and depression symptoms might lead individuals to be less connected to others [16].

In the sample from China and Greece, the factor “social connectedness” seemed to play a different role. In the sample from China, two sets of latent factors were identified: “objective” social participation, and “feeling” of connectedness. Perhaps the subtle differentiation between objective social participation and feeling the connectedness could mean merely reporting no problems was even further from positive outcomes for our Chinese sample, compared to other countries.

In Greece, the dominant factor found was social connectedness. It could be that social connectedness was valued differently in Greece than in other countries. Previous studies have found lower level of social connectedness in Greece compared to other countries [64–66].

### Risk factors

Apart from age, not living alone was positively associated with social connectedness. Perhaps even though social connectedness can be derived online [67], discouragements of physical contacts disrupt more sense of connection [68, 69]. Living with others could somewhat restore that sense.

### Strengths and limitations

The strengths of our study were that we collected data from eight countries, which varied in political orientation, social culture, and public health strategies. Second, anxiety, depression, self-confidence, and social connectedness were measured early in the COVID-19 pandemic in order to allow for follow-up at later stages. Third, we studied the correlations between anxiety, depression, self-confidence, and social connectedness; we used factor analysis to investigate which latent factors contributed highly to these aspects and compared these across countries.

Limitations of our study are mainly related to the new explorative instruments that were used, namely EQ-5D-5L bolt-on questions of social participation, contacts with family and friends, and feeling connected to others. Although EQ-5D bolt-on questions with similar psycho-social dimensions were examined in previous studies [70, 71], the bolt-on questions introduced in this study were new and not validated yet, apart from the self-confidence bolt-on. Further validation studies of social participation, contacts with family and friends, and feeling connected to others bolt-ons are required.

Second limitation is mainly related to sampling. First, the questionnaire was administrated online, and highly educated participants were overrepresented. Second, the sample sizes were similar between countries and does not match the population levels in each country. Third, the epidemiology profile of the COVID-19 differs greatly between countries at the time of this study. Additionally, we do not hold any information on non-response. These issues may limit the generalizability of our study.

Another limitation involved single-sourced measures. Independent and dependent variables in our study were collected from the same source. Although, prior to the study, we have carefully designed and worded the questionnaire and made sure that the independent and dependent variables were presented in separate modules of the questionnaire, Harman’s single factor test [72] suggested common method bias was present. This may affect the reliability and validity of the results.

## Conclusions

We conclude that anxiety, depression, and problems with social connectedness were highly prevalent in the general population of eight countries during the early phase of the COVID-19 pandemic. Given this high prevalence, practitioners and policymakers should highlight the importance of screening, especially for younger persons and females, and ensure that additional resources have been allocated to implement policies to mitigate the impact of the pandemic on mental health.

## Supplementary Information

**Additional file 1. **

## Data Availability

The dataset supporting the conclusions of this article is available for researchers who meet the criteria for access to data upon request which can be applied at the Data Access Committee of the Public Health Department (e-mail: secretariaat.mgz@erasmusmc.nl) under reference number 2022036.

## Abbreviations

POPCORN: Population health impact of the COVID-19 pandemic
UK: United Kingdom
US: United States
GAD-7: Generalized Anxiety Disorder-7
PHQ-9: Patient Health Questionnaire-9
WHO: World Health Organization
yrs.: Years
CN: China
GR: Greece
IT: Italy
NL: Netherlands
RU: Russia
SE: Sweden
