# Supplementary material for: Anxiety, depression, and social connectedness among the general population of eight countries during the COVID-19 pandemic
Source: Arch Public Health. 2022 Nov 18;80:237. doi: 10.1186/s13690-022-00990-4 (PMC9672616; doi:10.1186/s13690-022-00990-4)
Supplement: Supplementary file 1 — Additional file 1. [file 13690_2022_990_MOESM1_ESM.docx]

## **Anxiety, depression, and social connectedness among the general population of eight countries during the COVID-19 pandemic**

Di Long, Gouke J. Bonsel, Erica I. Lubetkin, Mathieu F. Janssen, Juanita A. Haagsma

# Appendix

Table and figures in the appendix:

Table 1. Percentage distribution of age, sex, and level of education among dropouts, compared to respondents in our study

Figure 1(A-H). Distribution of sex and age categories of the national population and our sample, by country

Table 2. Pandemic situation in each country at the start of data collection

Table 3. Spearman correlation between selected variables

Figure 2. Parallel analysis to determine the number of factors to consider on GAD-7 and PHQ-9 items

Table 4. Factor loading of factor analysis on GAD-7 and PHQ-9 items

#### Translations of questionnaires

The study questionnaire was translated from English into each country’s local language using translation software and then back translated into English. Bilingual native speakers verified the translations independently.

The questionnaire was translated into Simplified Chinese (China), Greek, Italian, Dutch (the Netherlands), Russian, Swedish (Sweden), English (UK), and English (US) respectively in China, Greece, Italy, the Netherlands, Russia, Sweden, the UK, and the US.

#### Categorizing levels of education and income

Based on the International Standard Classification of Education (ISCED-2011), the highest level of education achieved was categorized into three groups: up to lower secondary education (ISCED 0, 1 and 2; ‘low’), completed upper secondary education (ISCED 3 and 4; ‘mid') and tertiary education (ISCED 5 and above; ‘high').

Two types of data on income were collected. Monthly personal income (Greece and Russia) and annual household income (all other countries). Income was categorized from 12 groups into four groups: lower 20% (Q1), middle 60% (Q2), higher 20% (Q3), and prefer not to answer.

#### Response categories of Access of healthcare experienced

Two sets of response answers were used for the question on access of healthcare experienced. The ordinal response option of one set ranges from “very good” to “very bad” while that of the other set ranges from “always good” to “never good”. A random 50% of respondents answered the questionnaire with each set. The design of the 2-set response answers is part of an experiment on questionnaire wording and is irrelevant to our study. For the purpose of our study, ordinal response options were merged and ranged from “very good/always good” to “very bad/never good”.

#### Exclusion of respondents

46 respondents with sex “other” were excluded. 63 Greek respondents were excluded due to missing information on income. In our questionnaire, income categories in Greece were set unrealistic that most respondents (64%) reported the lowest category out of 12 categories. Existing information on income were collected from the panel member and we linked them with the unique ID to our data. However, 63 missing records were found, and we therefore excluded them from this study.

#### Dropout characteristics

2881 people started but did not finish the questionnaire (Table 1). Missing in the table means dropping out prior to completing this question.

Compared to completers, significant difference is marked by *. Dropouts were significantly younger in Russia and older in China, more likely to be female in Sweden and Russia, and differently distributed in level of education in all countries.

#### Data representativeness

To measure to what extent our data represent national data, the set of figures 1A-H were constructed. Respondents aged 18-, 19- and 75-years were excluded from the figures.

The percentage represents the percentage of that sex-age category out of the total population aged 20-74.

Overall, data representation is good. However, we observed that younger age males (20-29 years old) were underrepresented in many countries. Moreover, in China, older persons were underrepresented while younger females were overrepresented. In Greece, older females were underrepresented while younger females were overrepresented.

#### Internal consistency among instruments

After high Spearman correlation were found among several variables (Table 3), Cronbach’s alpha[1] was calculated among variables with related variables. Cronbach’s alpha is 0.765 among variables GAD-7 sum score, PHQ-9 sum score, and self-confidence, and 0.757 among variables “contact with family and friends”, “social participation”, and “feeling connected to others”.

#### Factor analysis on GAD-7 and PHQ-9 items

Because high correlations were found between GAD-7 and PHQ-9 sum scores in our results, we further performed exploratory factor analysis on the GAD-7 and PHQ-9 items in each country, in order to examine the structure behind GAD-7 and PHQ-9. The same procedure was followed to perform the exploratory factor analysis. Our data were tested to be suitable for factor analysis by Kaiser-Meyer-Olkin (KMO) measure of sampling adequacy and Bartlett’s test of Sphericity. Principal axis factoring was chosen as the extraction method. Because we were interested in the latent factor that underlines the data and that multivariate normality was violated in our data, the interrelationship was studied by principal axis factoring. The number of factors extracted was examined based on Scree test and parallel analysis. An oblique rotation (Promax rotation, kappa set at 4) was used. A set of factor matrix, pattern matrix, structure matrix, and factor correlation matrix were generated. If factors correlations were low, an orthogonal rotation would be chosen before re-run the rotation. Factor loading above 0.4 was considered high. The exploratory factor analysis was performed for each country and for all countries pooled, to compare if there were difference between countries in latent concepts behind the observed outcomes.

Parallel analysis suggested number of factors based on results value above 0. Four factors were suggested in each country (Figure 2). However, for several countries, parallel analysis results of the fourth factor were very low (≤0.07), after examining factor loadings (Pattern Matrix), the fourth factor was not included.

In most countries four factors were extracted expect for Greece, the UK and the US where three factor were considered (Figure 2). The dominant factor (F1) had eigenvalue from 7.5(Greece) to 10.3(US) and explained from 47.1% (Greece) to 64.4% (US). F1 had high influence on almost all GAD-7 items except for in China. Several less dominant factors (F2-F4) had high influence on different PHQ-9 items and one or two GAD-7 items. Almost all factors in each country were highly correlated with each other.

# Table and figures

#### Table 1. Percentage distribution of age, sex, and level of education among dropouts, compared to respondents in our study

|  |  | China % | | Greece % | | *Italy %* | | Netherlands % | |
| --- | --- | --- | --- | --- | --- | --- | --- | --- | --- |
|  |  | ***Dropout***  ***N=677*** | ***Completer***  ***N=3226*** | ***Dropout***  ***N=85*** | ***Completer***  ***N=959*** | ***Dropout***  ***N=144*** | ***Completer***  ***N=3210*** | ***Dropout***  ***N=489*** | ***Completer***  ***N=3293*** |
| % |  | 17.3% |  | 8.1% |  | 4.3% |  | 12.9% |  |
| Age | | * | |  |  |  |  |  |  |
|  | Median (IQR) | 40.0 (27.0) | 34.0(15.0) | 39.0 (21.0) | 39.0 (20.0) | 42.0 (24.3) | 43.0 (22.0) | 50.0 (31.0) | 49.0 (29.0) |
|  | Mean (SD) | 40.6 (15.7) | 35.9(11.7) | 39.1 (12.9) | 40.3 (13.2) | 41.7 (15.5) | 44.0 (14.2) | 48.4 (17.3) | 47.8 (16.6) |
| Sex | |  |  |  |  |  |  |  | |
|  | Male | 46.8% | 44% | 44.7% | 47.7% | 41.0% | 47.9% | 44.6% | 48.2% |
|  | Female | 52.3% | 56% | 55.3% | 52.3% | 59.0% | 52.1% | 55.4% | 51.8% |
| Education | | * | | * | |  | | * | |
|  | High | 38.7% | 59% | 45.9% | 61.2% | 43.1% | 41.5% | 53.6% | 44.4% |
|  | Middle | 55.5% | 31% | 50.6% | 34.9% | 33.3% | 44.5% | 26.4% | 30.4% |
|  | Low | 5.5% | 10% | 3.5% | 3.9% | 22.9% | 14.0% | 19.6% | 25.2% |
|  | Prefer not to answer | / | / | / | / | 0.4% | / | / | / |
|  | Missing | 0.1% | / | / | / | / | / | / | / |
|  |  | ***Russia %*** | | ***Sweden %*** | | ***UK %*** | | ***US %*** | |
|  |  | ***Dropout***  ***N=234*** | ***Completer***  ***N=3166*** | ***Dropout***  ***N=442*** | ***Completer***  ***N=3209*** | ***Dropout***  ***N=354*** | ***Completer***  ***N=3230*** | ***Dropout***  ***N=359*** | ***Completer***  ***N=3220*** |
| % |  | 6.9% |  | 12.2% |  | 9.9% |  | 10.0% |  |
| Age | | * | |  | |  | |  | |
|  | Median (IQR) | 36.0 (19.0) | 40.0 (23.0) | 47.0 (29.8) | 48.0(28.0) | 45.0 (29.0) | 44.0(27.0) | 46.0 (29.0) | 46.0(27.0) |
|  | Mean (SD) | 38.1 (13.3) | 40.7 (14.0) | 47.8 (16.8) | 47.6 (16.3) | 45.8 (16.8) | 45.5 (15.9) | 45.4 (16.6) | 46.5 (16.1) |
|  | Missing (#) | / | / | / | / | / | / | 8 | / |
| Sex | | * | | * | |  | |  | |
|  | Male | 41.5% | 50.2% | 39.1% | 47.3% | 44.1% | 48.2% | 38.2% | 43.9% |
|  | Female | 58.5% | 49.8% | 60.9% | 52.7% | 55.9% | 51.8% | 59.6% | 56.1% |
|  | Missing | / | / | / | / | / | / | 2.2% | / |
| Education | | * | | * | | * | | * | |
|  | High | 58.1% | 52.0% | 55.0% | 58.7% | 43.2% | 61.1% | 56.8% | 57.6% |
|  | Middle | 34.6% | 46.1% | 33.7% | 30.8% | 48.9% | 36.5% | 30.9% | 35.5% |
|  | Low | 6.8% | 1.9% | 11.1% | 10.5% | 7.9% | 2.3% | 12.3% | 6.9% |
|  | Missing | 0.4% | / | / | / | / | / | / | / |

#### Figure 1(A-H). Distribution of sex and age categories of the national population and our sample, by country


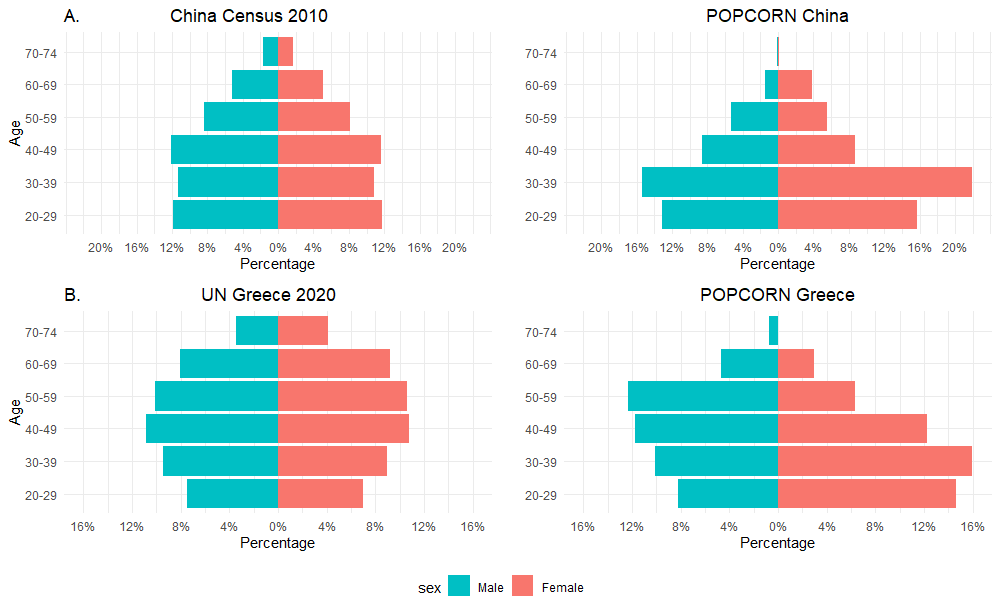


##
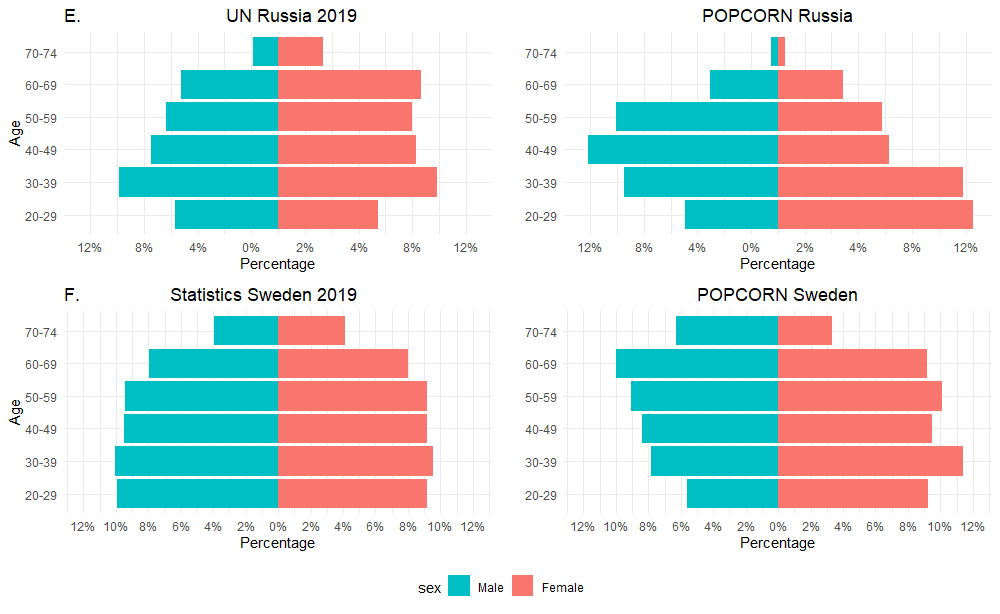

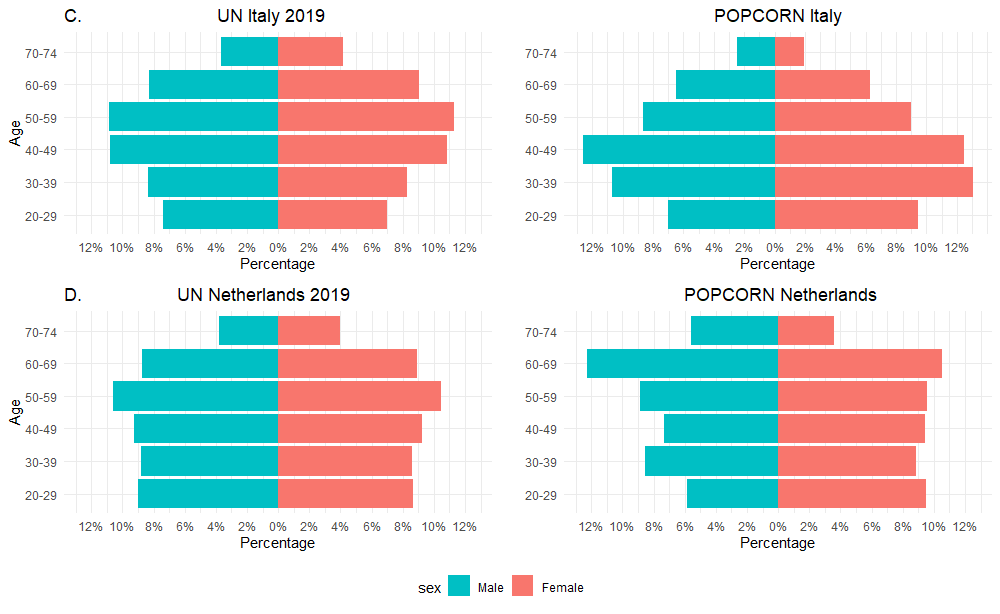

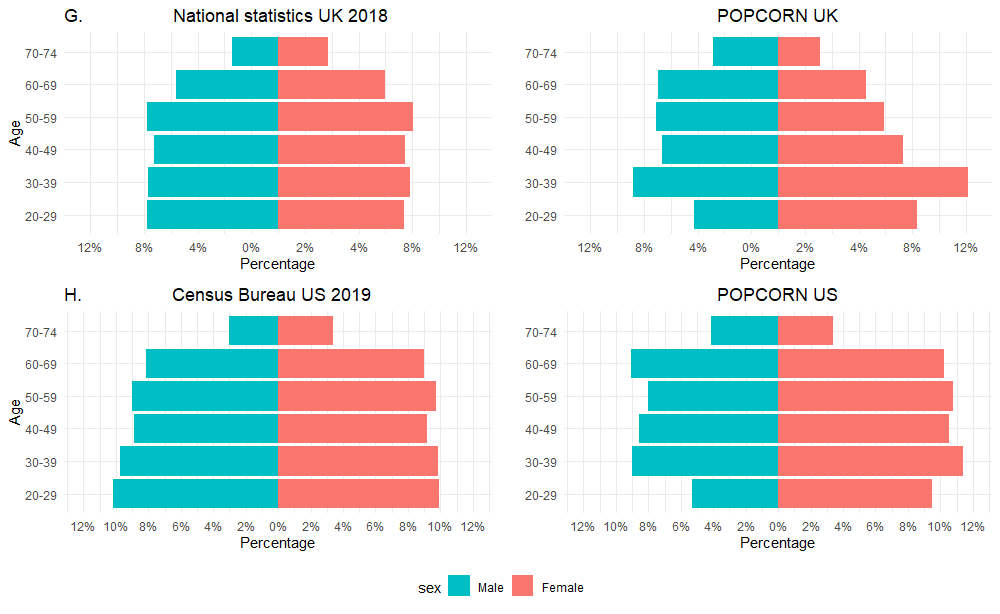


#### Table 2. Epidemiology of COVID-19 and government response against the spread of COVID-19, as measured by the stringency index in each country at the start of data collection

|  | **China** | **Greece** | **Italy** | **Netherlands** | **Russia** | **Sweden** | **UK** | **US** |
| --- | --- | --- | --- | --- | --- | --- | --- | --- |
| Start date of data collection | April 22^nd^ | April 22^nd^ | April 22^nd^ | April 22^nd^ | May 26^th^ | May 26^th^ | April 22^nd^ | April 22^nd^ |
| COVID-19 new cases per million people | 0.04 | 0.67 | 56.89 | 50.91 | 61.44 | 71.27 | 81.64 | 88.67 |
| COVID-19 cumulative cases per million people | 59.91 | 230.53 | 3162.15 | 2045.52 | 2497.14 | 3402.76 | 2093.96 | 2522.15 |
| COVID-19 new deaths per million people | 0.00 | 0.00 | 7.38 | 7.94 | 1.20 | 9.17 | 12.63 | 16.43 |
| COVID-19 cumulative death per million people | 3.25 | 3.54 | 423.44 | 232.26 | 26.24 | 394.09 | 314.32 | 148.75 |
| Stringency index | 56.94 | 84.26 | 93.52 | 78.70 | 78.24 | 64.81 | 79.63 | 72.69 |

Note to table: data source is Our World in Data (<https://ourworldindata.org/covid-cases>). Stringency index source: COVID-19 GOVERNMENT RESPONSE TRACKER[2, 3]

#### Table 3. Spearman correlation between selected variables

|  | Age | # Chronic disease | GAD-7 sum score | PHQ-9 sum score | Self-confidence | Contacts | Social participation |
| --- | --- | --- | --- | --- | --- | --- | --- |
| # Chronic disease | 0.184 |  |  |  |  |  |  |
| GAD-7 sum score | -0.249 | 0.301 |  |  |  |  |  |
| PHQ-9 sum score | -0.246 | 0.360 | ***0.822*** |  |  |  |  |
| Self-confidence | -0.226 | 0.276 | ***0.573*** | ***0.629*** |  |  |  |
| Social participation | -0.088 | 0.302 | 0.444 | 0.497 | ***0.503*** |  |  |
| Contacts | -0.007 | 0.237 | 0.404 | 0.448 | 0.434 | 0.441 |  |
| Feeling connected | -0.028 | 0.259 | 0.407 | 0.479 | ***0.503*** | ***0.505*** | ***0.552*** |

Note to table: all coefficients were significant at 0.01 level.

#### Figure 2. Parallel analysis to determine the number of factors to consider on GAD-7 and PHQ-9 items


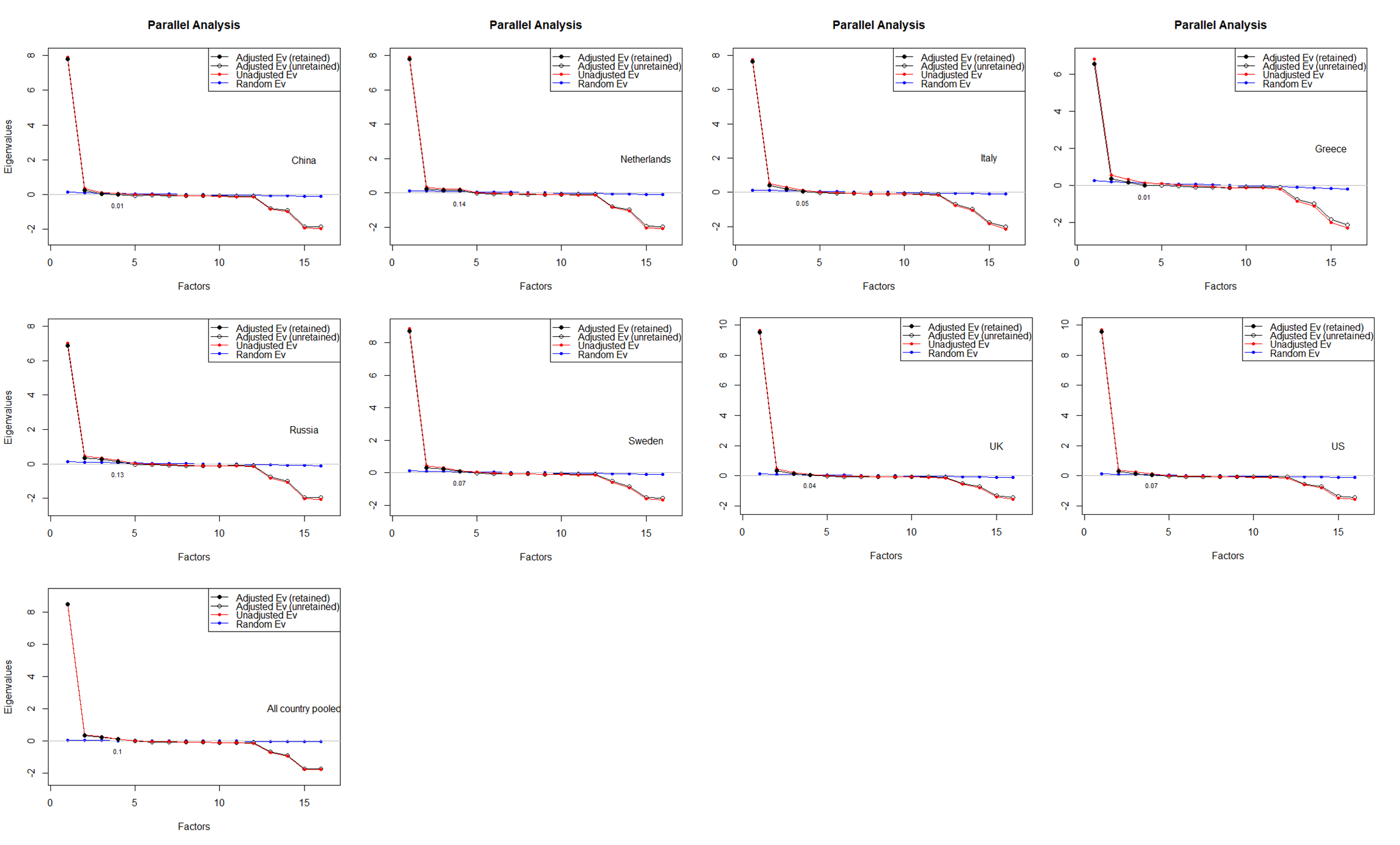


#### Table 4. Factor loading of factor analysis on GAD-7 and PHQ-9 items

|  | China | | | |  | Netherlands | | | |  | Italy | | | |  | Greece | | |
| --- | --- | --- | --- | --- | --- | --- | --- | --- | --- | --- | --- | --- | --- | --- | --- | --- | --- | --- |
|  | F1 | F2 | F3 | F4 |  | F1 | F2 | F3 | F4 |  | F1 | F2 | F3 | F4 |  | F1 | F2 | F3 |
| GAD-1: feeling nervous | **0.74** | 0.13 | -0.09 | 0.01 |  | **0.82** | 0.03 | -0.11 | 0.09 |  | **0.76** | 0.19 | -0.14 | -0.01 |  | **0.71** | 0.24 | -0.20 |
| GAD-2: not able to stop | **0.70** | -0.09 | 0.09 | 0.13 |  | **0.87** | -0.11 | 0.01 | 0.07 |  | **0.93** | -0.13 | 0.00 | 0.05 |  | **0.73** | -0.04 | 0.01 |
| GAD-3: worry too much | **0.79** | 0.11 | -0.05 | -0.05 |  | **0.86** | 0.04 | -0.08 | 0.04 |  | **0.90** | -0.11 | 0.02 | 0.07 |  | **0.79** | 0.07 | -0.10 |
| GAD-4: trouble relaxing | **0.65** | 0.08 | 0.15 | -0.08 |  | **0.65** | 0.25 | 0.09 | -0.10 |  | **0.72** | 0.23 | -0.05 | -0.09 |  | **0.78** | 0.11 | -0.05 |
| GAD-5: restless | 0.23 | -0.09 | **0.71** | -0.04 |  | **0.43** | 0.12 | 0.37 | -0.15 |  | **0.43** | 0.01 | **0.45** | -0.11 |  | **0.54** | -0.19 | **0.40** |
| GAD-6: easily annoyed | 0.34 | 0.33 | 0.09 | 0.04 |  | 0.33 | 0.24 | 0.14 | 0.08 |  | **0.48** | 0.26 | 0.08 | 0.00 |  | **0.66** | 0.10 | 0.03 |
| GAD-7: feeling afraid | 0.32 | 0.00 | **0.40** | 0.08 |  | **0.56** | -0.13 | 0.22 | 0.13 |  | **0.64** | -0.04 | 0.09 | 0.09 |  | **0.55** | -0.08 | 0.26 |
| PHQ-1: little interests | 0.23 | **0.51** | 0.05 | 0.03 |  | 0.10 | **0.40** | 0.00 | 0.33 |  | -0.01 | **0.67** | 0.01 | 0.14 |  | 0.06 | **0.69** | -0.06 |
| PHQ-2: feeling down | 0.18 | 0.29 | 0.10 | 0.29 |  | 0.28 | 0.19 | -0.07 | **0.53** |  | **0.42** | 0.26 | 0.01 | 0.29 |  | 0.25 | **0.50** | 0.14 |
| PHQ-3: trouble sleeping | 0.13 | **0.52** | 0.15 | -0.07 |  | 0.11 | **0.63** | 0.01 | 0.01 |  | 0.26 | **0.52** | -0.02 | -0.09 |  | 0.10 | **0.53** | 0.09 |
| PHQ-4: feeling tired | 0.15 | **0.80** | -0.05 | -0.11 |  | -0.07 | **0.88** | -0.04 | 0.01 |  | 0.02 | **0.81** | -0.02 | 0.00 |  | 0.00 | **0.75** | 0.09 |
| PHQ-5: poor appetite/overeat | -0.08 | 0.37 | **0.41** | 0.04 |  | -0.01 | 0.31 | 0.26 | 0.24 |  | 0.00 | **0.49** | 0.17 | 0.05 |  | 0.11 | 0.29 | 0.27 |
| PHQ-6: feeling bad about self | 0.12 | **0.41** | -0.02 | 0.29 |  | 0.10 | 0.09 | 0.07 | **0.59** |  | 0.08 | 0.19 | 0.27 | **0.42** |  | 0.10 | 0.32 | 0.33 |
| PHQ-7: trouble concentrating | 0.07 | 0.32 | 0.24 | 0.13 |  | 0.03 | 0.33 | **0.45** | 0.01 |  | 0.06 | 0.34 | 0.38 | 0.11 |  | -0.09 | 0.33 | **0.48** |
| PHQ-8: moving slow/fidgety | -0.14 | 0.15 | **0.63** | 0.13 |  | -0.05 | -0.02 | **0.73** | 0.08 |  | -0.02 | -0.01 | **0.86** | -0.02 |  | 0.08 | -0.07 | **0.71** |
| PHQ-9: better off dead | -0.02 | -0.08 | 0.03 | **0.80** |  | -0.01 | -0.16 | **0.45** | **0.52** |  | -0.08 | -0.01 | **0.59** | 0.29 |  | -0.17 | 0.12 | **0.63** |
| Eigenvalue | 8.6 | 1.0 | 0.7 | 0.6 |  | 8.8 | 0.9 | 0.8 | 0.7 |  | 8.6 | 1.2 | 0.8 | 0.6 |  | 7.5 | 1.3 | 0.9 |
| % Variance | 54.0 | 6.2 | 4.2 | 3.8 |  | 55.2 | 5.8 | 5.1 | 4.3 |  | 53.9 | 7.4 | 5.2 | 3.9 |  | 47.1 | 8.1 | 5.8 |
| Correlation between factors |  | F2 | F3 | F4 |  |  | F2 | F3 | F4 |  |  | F2 | F3 | F4 |  |  | F2 | F3 |
|  | F1 | 0.80 | 0.74 | 0.62 |  | F1 | 0.77 | 0.68 | 0.73 |  | F1 | 0.78 | 0.64 | 0.44 |  | F1 | 0.73 | 0.62 |
|  | F2 |  | 0.78 | 0.73 |  | F2 |  | 0.68 | 0.70 |  | F2 |  | 0.67 | 0.51 |  | F2 |  | 0.66 |
|  | F3 |  |  | 0.78 |  | F3 |  |  | 0.65 |  | F3 |  |  | 0.40 |  |  |  |  |

|  | Russia | | | |  | Sweden | | | |  | UK | | |  | US | | |  | All country pooled | | |
| --- | --- | --- | --- | --- | --- | --- | --- | --- | --- | --- | --- | --- | --- | --- | --- | --- | --- | --- | --- | --- | --- |
|  | F1 | F2 | F3 | F4 |  | F1 | F2 | F3 | F4 |  | F1 | F2 | F3 |  | F1 | F2 | F3 |  | F1 | F2 | F3 |
| GAD-1: feeling nervous | **0.86** | 0.07 | -0.13 | 0.03 |  | **0.88** | 0.09 | -0.02 | -0.08 |  | **0.91** | -0.12 | 0.05 |  | **0.93** | -0.12 | 0.03 |  | **0.84** | 0.14 | -0.14 |
| GAD-2: not able to stop | **0.78** | -0.15 | 0.07 | 0.09 |  | **0.87** | -0.09 | 0.09 | 0.04 |  | **0.97** | 0.04 | -0.11 |  | **0.92** | 0.03 | -0.05 |  | **0.89** | -0.10 | 0.07 |
| GAD-3: worry too much | **0.89** | -0.01 | -0.01 | -0.02 |  | **0.93** | -0.04 | 0.00 | 0.01 |  | **0.91** | -0.05 | 0.02 |  | **0.89** | -0.05 | 0.04 |  | **0.89** | 0.02 | -0.05 |
| GAD-4: trouble relaxing | **0.71** | 0.09 | 0.10 | -0.03 |  | **0.74** | 0.18 | -0.11 | 0.10 |  | **0.67** | 0.00 | 0.23 |  | **0.69** | 0.04 | 0.16 |  | **0.72** | 0.17 | -0.01 |
| GAD-5: restless | 0.27 | -0.04 | **0.65** | -0.18 |  | 0.31 | 0.03 | 0.02 | **0.52** |  | 0.38 | 0.37 | 0.09 |  | **0.52** | 0.37 | -0.05 |  | **0.42** | -0.04 | **0.42** |
| GAD-6: easily annoyed | **0.46** | 0.32 | 0.09 | -0.11 |  | **0.42** | 0.12 | 0.07 | 0.27 |  | **0.44** | 0.14 | 0.26 |  | **0.42** | 0.11 | 0.32 |  | **0.46** | 0.27 | 0.09 |
| GAD-7: feeling afraid | **0.56** | -0.04 | 0.13 | 0.15 |  | **0.53** | -0.04 | 0.14 | 0.23 |  | **0.66** | 0.20 | 0.01 |  | **0.63** | 0.20 | 0.03 |  | **0.61** | -0.01 | 0.22 |
| PHQ-1: little interests | -0.06 | **0.79** | -0.11 | 0.04 |  | 0.12 | 0.35 | 0.26 | -0.01 |  | 0.15 | 0.15 | **0.56** |  | 0.20 | 0.27 | 0.39 |  | 0.05 | **0.67** | 0.05 |
| PHQ-2: feeling down | 0.31 | 0.38 | -0.13 | 0.31 |  | 0.34 | 0.30 | 0.38 | -0.11 |  | **0.42** | 0.13 | 0.38 |  | 0.35 | 0.21 | 0.36 |  | 0.31 | **0.48** | 0.11 |
| PHQ-3: trouble sleeping | 0.10 | **0.55** | 0.04 | 0.01 |  | 0.07 | **0.76** | -0.09 | 0.06 |  | 0.06 | -0.07 | **0.79** |  | 0.12 | -0.09 | **0.76** |  | 0.12 | **0.66** | -0.03 |
| PHQ-4: feeling tired | 0.01 | **0.85** | 0.05 | -0.11 |  | -0.02 | **0.97** | -0.08 | -0.05 |  | -0.01 | -0.08 | **0.93** |  | -0.02 | -0.05 | **0.91** |  | -0.01 | **0.88** | -0.07 |
| PHQ-5: poor appetite/overeat | -0.03 | **0.44** | 0.25 | 0.08 |  | -0.03 | **0.40** | 0.32 | 0.15 |  | 0.06 | 0.38 | 0.36 |  | 0.00 | 0.38 | **0.43** |  | 0.01 | **0.44** | 0.32 |
| PHQ-6: feeling bad about self | 0.05 | 0.15 | -0.01 | **0.61** |  | 0.15 | 0.11 | **0.68** | -0.08 |  | 0.20 | 0.37 | 0.29 |  | 0.14 | 0.37 | 0.36 |  | 0.10 | 0.38 | 0.34 |
| PHQ-7: trouble concentrating | -0.04 | 0.17 | **0.43** | 0.27 |  | 0.07 | 0.24 | 0.30 | 0.28 |  | 0.08 | **0.52** | 0.29 |  | 0.11 | **0.54** | 0.22 |  | 0.07 | 0.27 | **0.49** |
| PHQ-8: moving slow/fidgety | -0.09 | -0.04 | **0.65** | 0.18 |  | -0.11 | -0.06 | **0.49** | **0.51** |  | -0.02 | **0.95** | -0.14 |  | 0.01 | **0.92** | -0.12 |  | -0.02 | -0.10 | **0.87** |
| PHQ-9: better off dead | 0.05 | -0.08 | 0.07 | **0.62** |  | 0.00 | -0.14 | **0.76** | 0.11 |  | -0.05 | **0.83** | -0.02 |  | -0.06 | **0.87** | -0.02 |  | -0.05 | 0.06 | **0.70** |
| Eigenvalue | 7.9 | 0.1 | 1.0 | 0.8 |  | 9.6 | 0.9 | 0.8 | 0.6 |  | 10.3 | 1.0 | 0.7 |  | 10.3 | 0.9 | 0.7 |  | 9.2 | 1.0 | 0.8 |
| % Variance | 49.3 | 7.0 | 6.4 | 5.1 |  | 60.2 | 5.8 | 5.2 | 3.7 |  | 64.1 | 6.0 | 4.3 |  | 64.4 | 5.7 | 4.3 |  | 58.0 | 6.2 | 5.1 |
| Correlation between factors |  | F2 | F3 | F4 |  |  | F2 | F3 | F4 |  |  | F2 | F3 |  |  | F2 | F3 |  |  | F2 | F3 |
|  | F1 | 0.73 | 0.65 | 0.66 |  | F1 | 0.78 | 0.74 | 0.66 |  | F1 | 0.76 | 0.82 |  | F1 | 0.77 | 0.81 |  | F1 | 0.80 | 0.73 |
|  | F2 |  | 0.62 | 0.74 |  | F2 |  | 0.74 | 0.60 |  | F2 |  | 0.78 |  | F2 |  | 0.79 |  | F2 |  | 0.75 |
|  | F3 |  |  | 0.62 |  | F3 |  |  | 0.66 |  |  |  |  |  |  |  |  |  |  |  |  |

[1] M. Tavakol and R. Dennick, "Making sense of Cronbach's alpha," *International journal of medical education,* vol. 2, p. 53, 2011.

[2] T. Hale *et al.*, "A global panel database of pandemic policies (Oxford COVID-19 Government Response Tracker)," *Nature human behaviour,* vol. 5, no. 4, pp. 529-538, 2021.

[3] T. Hale, S. Webster, A. Petherick, T. Phillips, and B. Kira, "Oxford COVID-19 government response tracker (OxCGRT)," *last updated,* vol. 8, p. 30, 2020.
